# Temporal trends in stroke-prevention medication use in patients with atrial fibrillation and chronic obstructive pulmonary disease

**DOI:** 10.3389/fphar.2026.1761934

**Published:** 2026-02-18

**Authors:** Chuan-Wei Shen, Nantawarn Kitikannakorn, Chung-Yu Chen, Buntitabhon Sirichanchuen, Rewadee Jenraumjit, Kuang-Ming Liao

**Affiliations:** 1 School of Pharmacy, Kaohsiung Medical University, Kaohsiung, Taiwan; 2 Department of Pharmaceutical Care, Faculty of Pharmacy, Chiang Mai University, Chiang Mai, Thailand; 3 Department of Pharmacy, Kaohsiung Medical University Hospital, Kaohsiung, Taiwan; 4 The Center for Medical and Health Technology Assessment (CM-HTA), Faculty of Pharmacy, Chiang Mai University, Chiang Mai, Thailand; 5 Master of Science Program (Mental Health), Multidisciplinary and Interdisciplinary School (MIdS), Chiang Mai University, Chiang Mai, Thailand; 6 Department of Internal Medicine, Chi Mei Medical Center, Chiali, Taiwan; 7 Department of Nursing, Min-Hwei Junior College of Healthcare Management, Tainan, Taiwan

**Keywords:** atrial fibrillation, chronic obstructive pulmonary disease, prescription pattern, stroke prevention, temporal trend

## Abstract

**Background:**

Patients with atrial fibrillation (AF) and concomitant chronic obstructive pulmonary disease (COPD) have been reported to exhibit a higher risk of stroke, particularly among those with a history of COPD exacerbations. However, the prescription patterns of stroke-prevention medications in this population remain unclear. This study aimed to characterize the temporal trends of stroke-prevention therapies among patients with COPD and AF, to describe concurrent trends in clinical outcomes, and to assess whether prescribing patterns differed by COPD exacerbation history.

**Methods:**

Newly diagnosed AF and COPD patients were identified from Taiwan’s National Health Insurance Research Database between 1 January 2013, and 31 December 2022. Prescription patterns of stroke-prevention medications within 1 year after AF diagnosis were examined. Stroke-prevention medications included warfarin, non–vitamin K antagonist oral anticoagulants (NOACs), and oral antiplatelet agents (OAPTs). The temporal changes of medication use and 1-year incidence rate of ischemic stroke and major bleeding across AF diagnosis years were analyzed.

**Results:**

A total of 13,072 patients were included. From 2013 to 2022, use of any NOAC increased from 15.4% to 65.4% of patients, whereas warfarin use declined from 24.0% to 6.1% (both p < 0.0001). For patient-level prescription patterns, the proportion of patients receiving NOAC only increased from 2.6% in 2013 to 33.1% in 2022. Use of NOAC plus OAPT also rose steadily from 7.6% to 29.6%, becoming the second most common pattern after 2018. The overall treatment landscape shifted markedly from warfarin- or antiplatelet-based regimens to those predominantly centered on NOACs (p < 0.0001). Concurrent trends in ischemic stroke and major bleeding incidence rates both declined significantly over time (p = 0.0025 and p < 0.0001, respectively). The prescription pattern showed no statistically significant difference between patients with or without a COPD exacerbation history.

**Conclusion:**

Nationwide real-world data demonstrated a dramatic shift over the past decade in stroke-prevention strategies among patients with coexisting COPD and AF, moving from warfarin- or OAPT-based therapy to predominantly NOAC-based therapy. Ischemic stroke and major bleeding were decreased over the calendar years. COPD exacerbation history did not alter stroke-prevention medication use, though this subgroup warrants attention due to elevated cardiovascular risks.

## 1 Introduction

Atrial fibrillation (AF), the most commonly diagnosed sustained cardiac arrhythmia, affects approximately 743 and 664 individuals per 100,000 population worldwide and in Asia (Linz et al., 2024), and is associated with an increased and heterogeneous risk of stroke depending on the presence of individual risk factors (Chao et al., 2024). Chronic obstructive pulmonary disease (COPD) affects cardiac rhythm through multiple interacting pathways involving functional and structural remodeling (Simons et al., 2021), and is related to a higher risk of incident AF (Liao and Chen, 2017; Grymonprez et al., 2019). Beyond its potential role in the development of AF, COPD may also adversely affect patients who already have AF. Compared to those with AF alone, patients with concomitant COPD have been observed to exhibit higher risks of ischemic stroke (Nadeem et al., 2015; Tsai et al., 2025) and bleeding events (Durheim et al., 2018; Rodríguez-Mañero et al., 2019). In addition, COPD exacerbations may contribute to subsequent cardiovascular diseases, including ischemic stroke (Simons et al., 2024).

Since 2009, the pivotal randomized controlled trials have established the efficacy and safety of non–vitamin K antagonist oral anticoagulants (NOACs) for stroke prevention in AF, leading to their widespread adoption as the cornerstone of stroke prevention (Joglar et al., 2024; Van Gelder et al., 2024). Despite the established efficacy of NOACs, data from the Global Anticoagulant Registry in the Field–Atrial Fibrillation (GARFIELD-AF), encompassing patients from multiple countries worldwide, indicated that around 30% of individuals with AF received antiplatelet agents alone or no stroke-prevention therapy (Steinberg et al., 2017; Fox et al., 2021). This finding highlights a substantial treatment gap between clinical evidence and real-world practice in stroke prevention among patients with AF.

In this context, although COPD may influence the pathophysiology, symptom burden, and therapeutic decision-making of AF, the impact of COPD on stroke-prevention medication use among patients with AF remains unclear. To date, no study has comprehensively characterized the prescription patterns of stroke-prevention therapies in AF patients with concomitant COPD. Prior investigations concluded in 2015 (Liao et al., 2023), underrepresenting contemporary practice amid the rapid uptake of NOACs, evolving guideline recommendations, and changing perceptions of ischemic stroke and bleeding risk in patients with COPD. A current, population-level assessment was therefore conducted among patients with AF concomitant with COPD to characterize temporal trends in the use of oral anticoagulants (OACs) and oral antiplatelet agents (OAPT), to investigate concurrent temporal trends in ischemic stroke and major bleeding, and to further examine whether prescribing trends differed according to COPD exacerbation history in this specific, high-risk population.

## 2 Materials and methods

### 2.1 Study design and data sources

A population-based retrospective study was conducted using the National Health Insurance Research Database (NHIRD) in Taiwan. The National Health Insurance program is a mandatory social welfare system that covers more than 99.6% of Taiwan’s population. Patients’ healthcare-seeking behaviors are recorded in this claims database, including visit dates, disease diagnoses, prescriptions, and medical procedures (Lin et al., 2018; Hsieh et al., 2019). Because patient identities in the database were de-identified and encrypted (Lin et al., 2018), the requirement for informed consent was waived. This study was approved by the Institutional Review Board (IRB) of Kaohsiung Medical University Hospital (IRB number: KMUHIRB-E(I)-20240010).

### 2.2 Study cohort

A two-step algorithm was applied to identify the target population of this study. The enrollment period was from 1 January 2013, to 31 December 2022. Patients were included if they had newly diagnosed COPD, defined as at least two outpatient visits or at least one inpatient or emergency visit with a COPD diagnosis [International Classification of Diseases, 9th Revision, Clinical Modification (ICD-9-CM): 490, 491, 492, 496; International Classification of Diseases, 10th Revision, Clinical Modification (ICD-10-CM): J40, J41, J42, J43, J44] during the enrollment period. In addition, eligible patients were required to have received at least one long-acting inhaler or one short-acting inhaler plus an oral methylxanthine, with all inhalers restricted to agents approved for COPD treatment in Taiwan, within 1 year after the first COPD diagnosis. Patients were excluded if they had missing or incomplete information in the Registry of Beneficiaries dataset; were younger than 40 years or older than 90 years; or had a history of asthma, lung cancer, or lung transplantation. Patients who had used COPD-related medications, as defined above, within 1 year prior to the first COPD diagnosis were also excluded.

The defined COPD cohort was then followed until the development of AF, defined as at least two outpatient visits or at least one inpatient or emergency visit with AF (ICD-9-CM: 427.3X; ICD-10-CM: I48.X) between 1 January 2013, and 31 December 2022. In addition, eligible patients were required to have received at least one antiarrhythmic drug prescription or to have undergone catheter ablation within 90 days of the first AF diagnosis. Patients were excluded if their first AF diagnosis was before their first COPD diagnosis. Those with less than 1 year of follow-up after the AF diagnosis due to death or enrollment termination were also excluded. The date of the first AF diagnosis in the selected population was defined as the index date. The diagnostic codes for COPD (Ho et al., 2018; Kuang et al., 2024) and AF (Jensen et al., 2012; Cozzolino et al., 2019; Yao et al., 2019) have been validated in previous studies and demonstrated robust validity.

### 2.3 Study outcomes and baseline characteristics

All subjects were followed for 1 year after the index date to identify prescriptions for any OAC (warfarin, dabigatran, rivaroxaban, apixaban, edoxaban) or any OAPT (aspirin, clopidogrel, ticagrelor, prasugrel, cilostazol, dipyridamole, ticlopidine). Patients prescribed any of these medications were classified into their respective categories, and two types of trend analyses were conducted based on this classification. The first analysis assessed temporal trends in the prescription proportions of each drug category (warfarin, any NOAC, any OAPT, and none) at the medication level, where none referred to patients who did not receive any stroke-prevention medication. In this approach, patients could contribute to multiple categories if they received prescriptions from more than one class of stroke-prevention medications during the observation period. The second analysis examined temporal trends at the patient level, categorizing each patient into mutually exclusive groups (warfarin, any NOAC, any OAPT, combinations of the above, or none) according to the medications they had received following their first AF diagnosis. Notably, the “combination” groups do not necessarily indicate concurrent use of multiple therapies; rather, they reflect patients who were prescribed more than one medication class at any point during the observation period, with prescriptions that may or may not overlap in time.

Within the defined COPD-AF cohort, ecological analyses were conducted to describe concurrent calendar-year trends in clinical outcomes, including ischemic stroke and major bleeding. Major bleeding comprised intracranial hemorrhage and gastrointestinal bleeding, whichever came first. The 1-year incidence rates were calculated at the calendar-year level according to the index year. Each subject was followed up until the outcome event or 1 year after the index date. Outcomes were ascertained using the first qualifying ICD-9-CM or ICD-10-CM diagnosis recorded after the index date (Supplementary Table S1).

In addition, patients were stratified into groups with and without COPD exacerbations history to further evaluate the potential influence of COPD severity on stroke-prevention medications in AF. COPD exacerbation was defined as a healthcare encounter with a primary diagnosis of COPD within 1 year prior to the index date, accompanied by a prescription for oral or parenteral antibiotics or steroids (Larsson et al., 2013; Wang et al., 2021). The definition of antibiotics was restricted to those recommended in the Taiwan guidelines for the treatment of pneumonia (Taiwan Society of Pulmonary and Critical Care Medicine, 2018).

Baseline characteristics were defined at the index date. Demographic variables included age and sex. Comorbidities were assessed within 1 year prior to the index date, including hypertension, diabetes, hyperlipidemia, heart failure, valvular heart diseases, peripheral arterial disease, ischemic heart disease, transient ischemic attack, ischemic stroke, systemic embolism, gastrointestinal bleeding, intracranial hemorrhage, chronic kidney disease, chronic liver disease, sleep apnea, hyperthyroidism, percutaneous coronary intervention (PCI), coronary artery bypass graft (CABG), and CHA_2_DS_2_-VASc score categories. Comorbidities were defined based on at least three outpatient diagnoses or at least one inpatient/emergency diagnosis.

### 2.4 Statistical analyses

Continuous variables were presented as means and standard deviations (SD), whereas categorical variables were presented as numbers and percentages. For the distribution of variables between groups with or without a history of COPD exacerbation, an absolute standardized mean difference (ASMD) ≤ 0.1 was considered to indicate adequate balance between the two groups. Temporal changes in medication groups across index years were evaluated through trend analyses. The Cochran–Armitage test was applied for binary categorical variables, whereas the Cochran–Mantel–Haenszel test was used for categorical variables with more than two levels.

The 1-year incidence rates of clinical outcomes (per 100 person-years) and corresponding 95% confidence intervals (95% CIs) were estimated using Poisson regression with an offset for the log of person-years for each index year. The incidence rate ratio (IRR) increased per 1 year was assessed by modeling the index year as a continuous variable in Poisson regression; the p-values from the Wald test of the index-year coefficient were used to describe the statistical significance of temporal trends of incidence rates.

We fitted logistic regression models with cubic splines for calendar index year and tested group differences, defined by the presence or absence of a history of COPD exacerbation, by including an interaction term between group and spline terms. The global p-values for the interaction were examined by Wald chi-square test and used to assess whether the temporal trends differed between groups. All statistical analyses were conducted using SAS version 9.4 (SAS Institute Inc., Cary, NC, United States). Two-sided p-values ≤0.05 were considered statistically significant.

## 3 Results

**FIGURE 1:**
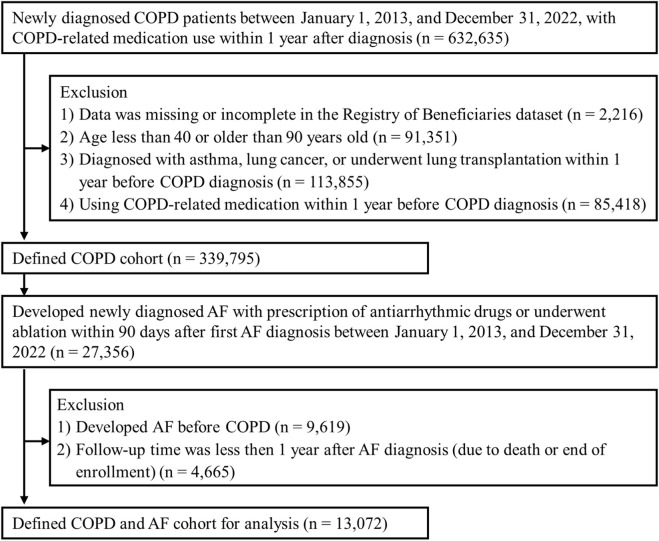
Flow chart of COPD and AF patients selection. AF: atrial fibrillation; COPD: chronic obstructive pulmonary disease.

A total of 632,635 patients were enrolled between 1 January 2013, and 31 December 2022. During follow-up, 27,356 individuals within the defined COPD cohort (n = 339,795) developed AF based on the predefined criteria. After excluding patients whose AF diagnosis occurred prior to their COPD diagnosis and patients with less than 1 year of follow-up, a total of 13,072 patients remained eligible for the final prescription pattern analysis. The cohort selection process is illustrated in Figure 1.

In the study population, the mean (SD) age was 75.0 (10.2) years, and the majority of patients were male (n = 9,066; 69.4%). The most prevalent comorbidities included hypertension (n = 8,898; 68.1%), diabetes (n = 4,007; 30.7%), hyperlipidemia (n = 3,220; 24.6%), heart failure (n = 4,727; 36.2%), and ischemic heart disease (n = 4,881; 37.3%). Over 87% of population accompanied with a CHA_2_DS_2_-VASc score ≥2, reflecting a moderate-to-high baseline ischemic stroke risk in this population. Overall, most baseline characteristics were well balanced between patients with and without a history of COPD exacerbation. However, notable imbalances were observed in sex distribution (male: 75.4% vs. 67.5%; ASMD = 0.18) and the prevalence of heart failure (41.1% vs. 34.7%; ASMD = 0.13). Detailed baseline characteristics for both groups are presented in Table 1.

**TABLE 1:** Baseline characteristics of defined COPD and AF cohort.

Characteristics	Total (n = 13,072)	With COPD exacerbation (n = 3,045)	Without COPD exacerbation (n = 10,027)	ASMD^a^
Demographics				
Mean age at AF (SD), year	75.0 (10.2)	75.7 (10.0)	74.8 (10.3)	0.09
Male, n (%)	9,066 (69.4)	2,297 (75.4)	6,769 (67.5)	0.18
Comorbid conditions in preceding 1 year, n (%)				
Hypertension	8,898 (68.1)	2,119 (69.6)	6,779 (67.6)	0.04
Diabetes	4,007 (30.7)	907 (29.8)	3,100 (30.9)	0.03
Hyperlipidemia	3,220 (24.6)	701 (23.0)	2,519 (25.1)	0.05
Heart failure	4,727 (36.2)	1,252 (41.1)	3,475 (34.7)	0.13
Valvular heart disease	684 (5.2)	183 (6.0)	501 (5.0)	0.04
Peripheral arterial disease	332 (2.5)	74 (2.4)	258 (2.6)	0.01
Ischemic heart disease	4,881 (37.3)	1,108 (36.4)	3,773 (37.6)	0.03
Transient ischemic attack	421 (3.2)	97 (3.2)	324 (3.2)	<0.01
Ischemic stroke	1,429 (10.9)	295 (9.7)	1,134 (11.3)	0.05
Systemic embolism	165 (1.3)	34 (1.1)	131 (1.3)	0.02
Gastrointestinal bleeding	1,509 (11.5)	366 (12.0)	1,143 (11.4)	0.02
Intracranial hemorrhage	445 (3.4)	90 (3.0)	355 (3.5)	0.03
Chronic kidney disease	2,454 (18.8)	532 (17.5)	1,922 (19.2)	0.04
Chronic liver disease	1,119 (8.6)	217 (7.1)	902 (9.0)	0.07
Sleep apnea	81 (0.6)	24 (0.8)	57 (0.6)	0.03
Hyperthyroidism	216 (1.7)	45 (1.5)	171 (1.7)	0.02
Percutaneous coronary intervention	856 (6.5)	189 (6.2)	667 (6.7)	0.02
Coronary artery bypass surgery	129 (1.0)	38 (1.3)	91 (0.9)	0.03
Mean CHA_2_DS_2_-VASc score (SD)	3.5 (1.7)	3.5 (1.7)	3.5 (1.7)	0.01
CHA_2_DS_2-_VASc score classification, n (%)				
Male in 0 and female in 1	613 (4.7)	124 (4.1)	489 (4.9)	0.05
Male in 1	1,067 (8.2)	241 (7.9)	826 (8.2)	​
Male or female ≥2	11,392 (87.2)	2,680 (88.0)	8,712 (86.9)	​

^a^ An absolute standardized mean difference ≤0.1 indicates a negligible difference in means or percentages between two groups.

**FIGURE 2:**
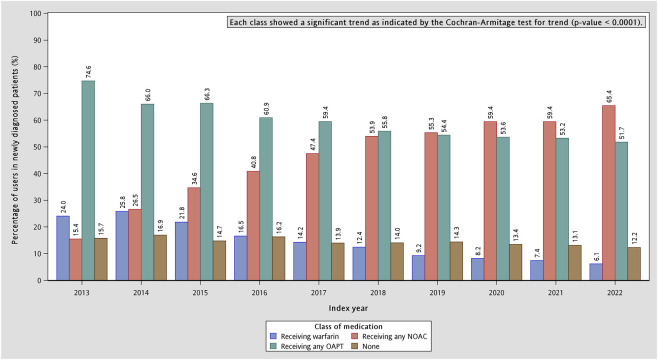
Temporal trends in the prescription proportions of stroke-prevention medication categories in COPD and AF patients. AF: atrial fibrillation; COPD: chronic obstructive pulmonary disease; NOAC: non-vitamin K antagonist oral anticoagulant; OAPT: oral antiplatelet agent.

The temporal trends in the prescription patterns of stroke-prevention medications among patients with COPD and AF from 2013 to 2022 is shown in Figure 2. In 2013, OAPTs were the predominant option for stroke prevention (74.6%), followed by warfarin (24.0%), whereas the use of NOACs was relatively limited (15.4%). Over time, NOAC prescriptions increased substantially, rising from 15.4% in 2013 to 65.4% in 2022, and rapidly overtook warfarin, which declined steadily to 6.1% by 2022. The overall proportion of patients receiving any OAPT decreased modestly, from 74.6% in 2013 to 51.7% in 2022. The temporal trends in all drug categories were statistically significant (p-value for trend <0.0001).

**FIGURE 3:**
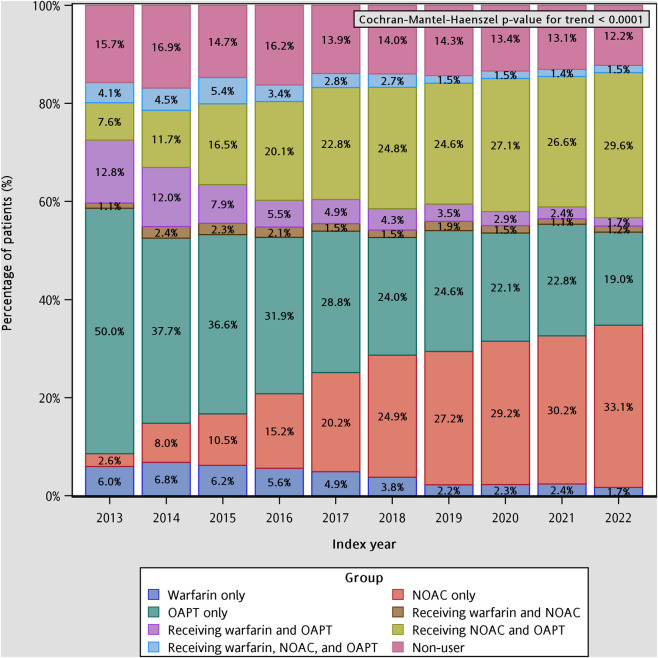
Temporal trends in the patient-level prescription pattern of stroke-prevention medications in COPD and AF patients. AF: atrial fibrillation; COPD: chronic obstructive pulmonary disease; NOAC: non-vitamin K antagonist oral anticoagulant; OAPT: oral antiplatelet agent.

As shown in Figure 3, patient-level prescription patterns of stroke-prevention therapies demonstrated substantial changes from 2013 to 2022. In 2013, half of the patients with COPD and AF (50.0%) received OAPT only within 1 year after their AF diagnosis, making it the most common regimen. Receiving warfarin plus OAPT was the second most frequent pattern (12.8%), followed by NOAC plus OAPT (7.6%) and warfarin only (6.0%). Non-users accounted for 15.7% of the cohort in 2013. Marked shifts occurred over the study period. The proportion of patients receiving NOAC only increased more than tenfold, rising from 2.6% in 2013 to 33.1% in 2022. Similarly, NOAC plus OAPT demonstrated a steady and substantial increase from 7.6% to 29.6%, and had become the second most common regimen after 2018. In contrast, use of warfarin plus OAPT exhibited a progressive decline from 12.8% in 2013 to 1.7% in 2022, while warfarin only also decreased from 6.0% to 1.7% over the period. The proportion of patients receiving OAPT only dropped sharply from 50.0% to 19.0%, reflecting a shift away from antiplatelet-based strategies. The prevalence of NOAC-and-warfarin pattern remained consistently low (<5% annually), and receiving three kinds of stoke-prevention medications (warfarin + NOAC + OAPT) remained a rare practice throughout the study window. The proportion of non-users decreased modestly from 15.7% to 12.2%, suggesting improved adoption of stroke-prevention therapies over time. The Cochran–Mantel–Haenszel trend test confirmed significant temporal changes across all treatment categories (p < 0.0001), highlighting a major and sustained shift from warfarin- and OAPT-centered regimens toward NOAC-dominant therapeutic strategies.

**FIGURE 4:**
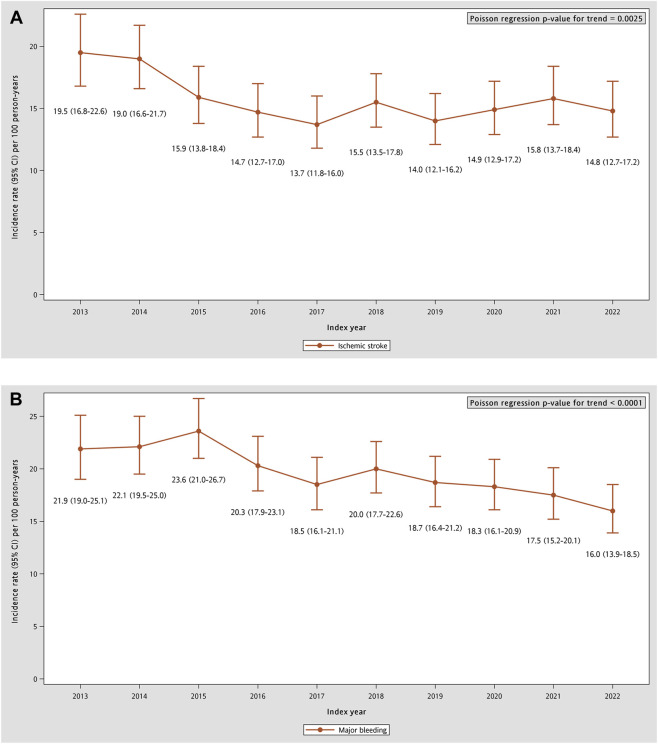
Temporal trends of 1-year incidence rates of clinical outcomes in COPD and AF patients. **(A)** Ischemic stroke. **(B)** Major bleeding, composed of intracranial hemorrhage and gastrointestinal bleeding. AF: atrial fibrillation; COPD: chronic obstructive pulmonary disease.

From 2013 to 2022, the annual distribution of stroke-prevention medications shifted substantially toward NOAC-based therapy. Over the same period, population-level incidence rates of ischemic stroke and major bleeding declined significantly (Figure 4). The 1-year ischemic stroke incidence rate (95% CI) decreased from 19.5 (16.8–22.6) per 100 person-years in 2013 to 14.8 (12.7–17.2) in 2022 (IRR 0.975, 95% CI 0.959–0.991; p = 0.0025). Major bleeding (95% CI) similarly fell from 21.9 (19.0–25.1) to 16.0 (13.9–18.5) per 100 person-years (IRR 0.965, 95% CI 0.951–0.979; p < 0.0001).

**FIGURE 5:**
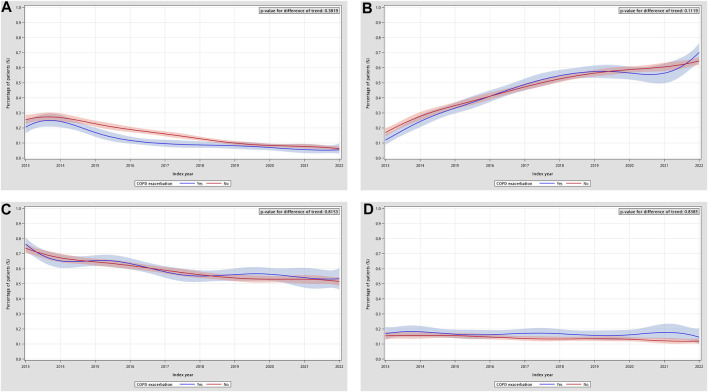
Temporal trends in the prescription proportions of stroke-prevention medication categories in COPD and AF patients, separated by COPD exacerbation history. **(A)** Trend of warfarin users by COPD exacerbation. **(B)** Trend of NOAC users by COPD exacerbation. **(C)** Trend of OAPT users by COPD exacerbation. **(D)** Trend of non-users by COPD exacerbation. AF: atrial fibrillation; COPD: chronic obstructive pulmonary disease; NOAC: non-vitamin K antagonist oral anticoagulant; OAPT: oral antiplatelet agent.

During the follow-up period, no statistically significant differences were observed between patients with and without a history of COPD exacerbation in the temporal trends of drug prescriptions. The trend comparison between the two groups showed no significant divergence in the trajectories of warfarin (p = 0.3819) or NOAC use (p = 0.1119). Similarly, the temporal patterns of OAPT use (p = 0.8153) and non-user proportions (p = 0.8385) did not differ significantly between the two subgroups (Figure 5). These findings suggested that although the overall directions of change differed across medication classes, the temporal trends were similar between patients with and without prior COPD exacerbations.

## 4 Discussion

Using a nationwide claims database in Taiwan, this cohort study investigated the prescription patterns of stroke-prevention medications and their temporal changes in recent years among COPD patients with newly diagnosed AF in the real-world clinical settings.

During the 10-year enrollment period, we found that approximately 8.1% of patients with general COPD had comorbid AF, which is consistent with the finding of previous study of 4.7%–15.0% (Papaporfyriou et al., 2023). Xiao et al. also observed prevalence of AF was up to 18.1% among patients with end-stage COPD on home oxygen who were hospitalized for COPD exacerbation (Xiao et al., 2019). On the other hand, we reported 5.2% incident cases of AF patients diagnosed after COPD, whereas a 9.8% newly diagnosed AF was reported in the COPD cohort observed by Liao et al. (Liao and Chen, 2017). The lower incidence in our study may reflect stricter eligibility criteria—including medication and procedure codes related to arrhythmia—that likely improved AF case ascertainment while restricting the eligible population.

Although several studies have examined stroke-prevention prescribing patterns in patients with AF and comorbid COPD, their findings have been inconsistent and have not provided a comprehensive characterization of the overall prescribing landscape in this population. In the Global Registry on Long-Term Antithrombotic Treatment in Patients with Atrial Fibrillation (GLORIA-AF) registry, AF patients with comorbid COPD had higher odds of receiving OACs than those without COPD (adjusted odds ratio 1.29, 95% CI 1.13–1.47) (Romiti et al., 2023). By contrast, the Outcomes Registry for Better Informed Treatment of Atrial Fibrillation (ORBIT-AF) reported similar OACs use between AF patients with and without COPD (76.1% vs. 76.4%, p = 0.763) (Durheim et al., 2018). These divergent findings may reflect differences in geographic coverage (GLORIA-AF is multinational, whereas ORBIT-AF is American-based), study design as well as eligibility criteria, and local practices (Huisman et al., 2014; Steinberg et al., 2014).

Liao et al. conducted the study using Taiwan’s NHIRD and reported that 31.1% of patients were prescribed low-dose aspirin within the first 30 days after AF diagnosis, whereas only a limited proportion received dabigatran (1.7%) or rivaroxaban (1.6%) (Liao et al., 2023). However, the enrollment period in Liao et al.‘s study was 2002–2015. To our knowledge, the reimbursement for AF-related stroke prevention under Taiwan’s National Health Insurance was introduced in the following years: dabigatran (2012), rivaroxaban (2013), apixaban (2014), and edoxaban (2016). Accordingly, our study can be considered an essential extension and refinement of their work. By incorporating data from more recent years, our study captured the contemporary landscape of stroke-prevention medications—reflecting reimbursement-driven uptake of NOACs and evolving practice patterns after 2016—thereby providing a more complete picture of prescribing in COPD patients with AF. However, because we did not include a contemporaneous AF cohort without COPD, we could not directly quantify whether COPD status modified the pace of NOACs adoption beyond temporal trends in AF management during 2013–2022. Accordingly, our findings should be interpreted as descriptive temporal trends among patients with AF and COPD. Nevertheless, quantifying real-world prescription pattern in this high-risk and clinically complex population remains meaningful, as COPD is frequently accompanied by greater comorbidity burden and treatment complexity.

With respect to the overall AF population—regardless of COPD status—several registry cohorts have reported the use of stroke-prevention medications; however, gaps remain in the literature, particularly for the most recent years. GARFIELD-AF, which recruited over 50,000 AF patients from 2010 to 2016 across 35 countries, showed that NOAC use, with or without OAPT, increased substantially during follow-up (from 3% in 2010 to 43% in 2016) (Steinberg et al., 2017; Kakkar et al., 2012). In our study, a similar trend was observed, revealing that 10.2% of patients using NOAC with or without OAPT in 2013, increasing to 35.3% in 2016, and 62.7% in 2022. Compared with the ORBIT-AF launched in the United States, the penetration of NOAC in Taiwan was relatively lower (71% vs. 35.3% in 2016) (Steinberg et al., 2017; Steinberg et al., 2014), which may be attributed to the higher bleeding risk reported in Asian or Chinese populations (Kim et al., 2021). As a result, OAPT was more frequently adopted as the preferred treatment option (Chiang et al., 2015).

Incorporating ecological outcome trends provides clinical context for the pronounced transition from warfarin- or OAPT-based strategies to NOACs in this high-risk COPD-AF population. At the population level, incidence rates of ischemic stroke and major bleeding declined over time, in parallel with the period of increasing NOAC uptake. However, these population-level associations should be interpreted cautiously. Because patient risk profiles, diagnostic coding practices, and other aspects of care changed concurrently over time, this ecological analysis cannot directly attribute outcome changes to NOAC adoption or infer causal treatment effects.

An exacerbation of COPD can precipitate both AF and ischemic stroke through converging pathways. Ventilation–perfusion mismatch with hypoxemia creates an oxygen supply–demand imbalance and, together with oxidative stress, dynamic hyperinflation, and sympathetic activation, promotes atrial remodeling and autonomic instability, heightening susceptibility to AF. In parallel, intercurrent infection and systemic inflammation drive endothelial dysfunction, platelet activation, and coagulation cascade upregulation, fostering a hypercoagulable status that predisposes to ischemic stroke (Simons et al., 2024). Beyond mechanistic plausibility, both moderate and severe COPD exacerbations have been observed to be associated with subsequent cardiovascular disease (CVD), with incidence peaking up to 20-fold hazard in the early post-exacerbation period (e.g., within weeks) and then gradually declining, yet remaining significantly elevated for up to 1 year after the exacerbation (Dransfield et al., 2022; Swart et al., 2023; Hawkins et al., 2024). However, in our analysis stratified by COPD exacerbation history, the post-AF prescribing trends for stroke-prevention medications did not differ significantly; even the prescribing proportions were similar, as suggested by the interweaving curves (Figure 5). Several factors may underlie these observations. First, clinical awareness of the implications of COPD exacerbations may remain heterogeneous, and current understanding may be insufficient to influence downstream prescribing decisions. Second, in our baseline assessment, patients with and without prior exacerbations exhibited similar CHA_2_DS_2_-VASc score distributions, suggesting generally guideline-concordant practice in Taiwan, which might overshadow the specific impact of COPD exacerbations on stroke-prevention medications choice. Third, according to clinical guidelines, COPD exacerbations are characterized by increased respiratory symptoms and require specific clinical assessments (GOLD Executive Committee, 2025). However, such detailed evaluations cannot be fully captured in claims-based data. Our findings may reflect the lack of granular clinical data on COPD exacerbation characteristics (e.g., laboratory assessments such as blood eosinophil counts and C-reactive protein, or specific triggers). In this study, we used COPD primary diagnosis in combination with prescriptions of oral or parenteral antibiotics or steroids as a proxy indicator to classify whether patients had a history of exacerbations (Larsson et al., 2013; Wang et al., 2021), which may underestimate the occurrence of COPD exacerbations. Nevertheless, given prior evidence that COPD exacerbations substantially increase subsequent CVD risk, this population still warrants particular attention to ensure comprehensive care.

### 4.1 Strengths and limitations of this study

The national health claims database in Taiwan provides detailed records of medical services for all citizens, which strengthens our ability to depict the overall treatment landscape. In addition, our cohort definitions have been well established in prior studies. We further applied medication and procedure records to confirm case identification.

Two limitations of this study should be specified. Firstly, patients with concomitant COPD and AF frequently present with a high burden of cardiovascular comorbidities that are associated with multifactorial thromboembolism risk; for example, heart failure was common in our cohort (36.2%). In this setting, clinical decision-making often involves multiple concurrent guideline-directed therapies. For instance, beta blockers may be lower in the COPD population due to concerns regarding bronchospasm, which could plausibly influence downstream cardiovascular outcomes, including ischemic stroke risk, through mechanisms not directly attributable to AF-related stroke. As a real-world, claims-based survey, however, we were unable to exhaustively exclude all non-AF pathways to stroke or fully disentangle the indications driving antithrombotic prescribing (e.g., concomitant coronary or peripheral arterial disease). Accordingly, our findings, framed exclusively around “stroke-prevention” medications, may not be fully attributable to the joint impact of COPD and AF, because the observed prescribing patterns may also reflect treatment decisions that intersect with other guideline-directed therapies and therefore may not fully represent the multidimensional cardiovascular risk profile of patients with concomitant AF and COPD. Secondly, The NHIRD lacks information required to define COPD severity, such as spirometry measures (e.g., forced expiratory volume in 1 second [FEV_1_]) and smoking status/history. In addition, our claims database does not capture the acute symptom changes or laboratory findings that characterize COPD exacerbations. Therefore, our observed lack of differences in stroke-prevention prescribing patterns by COPD exacerbation history may reflect limitations in exposure definition or a gap between pathophysiological risk and real-world prescribing behavior, and should not be interpreted as implying that COPD exacerbations are clinically irrelevant to cardiovascular management.

## 5 Conclusion

The complete treatment patterns and temporal trends of stroke-prevention medications in patients with COPD and AF were examined using data up to the most recent available year. By incorporating contemporary data spanning the past decade, we observed a clear shift from OAPT- or warfarin-based regimens—which previously constituted the backbone of stroke prevention—to a predominant reliance on NOACs in recent years. This transition mirrors the prescribing patterns reported in the general AF population, suggesting that the evolution of clinical practice toward NOAC-centered therapy has similarly extended to patients with concomitant COPD. Concurrent trends in ischemic stroke and major bleeding incidence rates were examined alongside prescribing trends; both clinical outcomes declined significantly over time. Although a history of COPD exacerbation did not appear to be associated with the use of stroke-prevention medications, this subgroup warrants close attention given the markedly increased risks of CVD and stroke following exacerbations.

## Data Availability

Data are available from the National Health Insurance Research Database (NHIRD) published by Taiwan National Health Insurance Administration. Due to legal restrictions imposed by the government of Taiwan in relation to the “Personal Information Protection Act”, data cannot be made publicly available. Requests for data can be sent as a formal proposal to the NHIRD (http://nhird.nhri.org.tw).

## References

[B1] Chao T. F. Potpara T. S. Lip G. Y. H. (2024). Atrial fibrillation: stroke prevention. Lancet Reg. Health Eur. 37, 100797. 10.1016/j.lanepe.2023.100797 38362551 PMC10867001

[B2] Chiang C. E. Wang K. L. Lin S. J. (2015). Asian strategy for stroke prevention in atrial fibrillation. Europace 17, ii31–ii39. 10.1093/europace/euv231 26842113

[B3] Cozzolino F. Montedori A. Abraha I. Eusebi P. Grisci C. Heymann A. J. (2019). A diagnostic accuracy study validating cardiovascular ICD-9-CM codes in healthcare administrative databases. The Umbria data-value project. PLoS One 14, e0218919. 10.1371/journal.pone.0218919 31283787 PMC6613689

[B4] Dransfield M. T. Criner G. J. Halpin D. M. G. Han M. K. Hartley B. Kalhan R. (2022). Time-dependent risk of cardiovascular events following an exacerbation in patients with chronic obstructive pulmonary disease: post hoc analysis from the IMPACT trial. J. Am. Heart Assoc. 11, e024350. 10.1161/jaha.121.024350 36102236 PMC9683674

[B5] Durheim M. T. Holmes D. N. Blanco R. G. Allen L. A. Chan P. S. Freeman J. V. (2018). Characteristics and outcomes of adults with chronic obstructive pulmonary disease and atrial fibrillation. Heart 104, 1850–1858. 10.1136/heartjnl-2017-312735 29875139

[B6] Fox K. A. A. Virdone S. Pieper K. S. Bassand J. P. Camm A. J. Fitzmaurice D. A. (2021). GARFIELD-AF risk score for mortality, stroke, and bleeding within 2 years in patients with atrial fibrillation. Eur. Heart J. Qual. Care Clin. Outcomes 8, 214–227. 10.1093/ehjqcco/qcab028 33892489 PMC8888127

[B7] GOLD Executive Committee (2025). Global strategy for the diagnosis, management, and prevention of chronic obstructive pulmonary disease. Available online at: https://goldcopd.org/(Accessed October 25, 2025).

[B8] Grymonprez M. Vakaet V. Kavousi M. Stricker B. H. Ikram M. A. Heeringa J. (2019). Chronic obstructive pulmonary disease and the development of atrial fibrillation. Int. J. Cardiol. 276, 118–124. 10.1016/j.ijcard.2018.09.056 30268382

[B9] Hawkins N. M. Nordon C. Rhodes K. Talukdar M. McMullen S. Ekwaru P. (2024). Heightened long-term cardiovascular risks after exacerbation of chronic obstructive pulmonary disease. Heart 110, 702–709. 10.1136/heartjnl-2023-323487 38182279 PMC11103306

[B10] Ho T. W. Ruan S. Y. Huang C. T. Tsai Y. J. Lai F. Yu C. J. (2018). Validity of ICD9-CM codes to diagnose chronic obstructive pulmonary disease from national health insurance claim data in Taiwan. Int. J. Chron. Obstruct Pulmon Dis. 13, 3055–3063. 10.2147/copd.S174265 30323577 PMC6174682

[B11] Hsieh C. Y. Su C. C. Shao S. C. Sung S. F. Lin S. J. Kao Yang Y. H. (2019). Taiwan's national health insurance research database: past and future. Clin. Epidemiol. 11, 349–358. 10.2147/clep.S196293 31118821 PMC6509937

[B12] Huisman M. V. Lip G. Y. Diener H. C. Dubner S. J. Halperin J. L. Ma C. S. (2014). Design and rationale of global registry on long-term oral antithrombotic treatment in patients with atrial fibrillation: a global registry program on long-term oral antithrombotic treatment in patients with atrial fibrillation. Am. Heart J. 167, 329–334. 10.1016/j.ahj.2013.12.006 24576516

[B13] Jensen P. N. Johnson K. Floyd J. Heckbert S. R. Carnahan R. Dublin S. (2012). A systematic review of validated methods for identifying atrial fibrillation using administrative data. Pharmacoepidemiol Drug Saf. 21 (Suppl. 1), 141–147. 10.1002/pds.2317 22262600 PMC3674852

[B14] Joglar J. A. Chung M. K. Armbruster A. L. Benjamin E. J. Chyou J. Y. Cronin E. M. (2024). ACC/AHA/ACCP/HRS guideline for the diagnosis and management of atrial fibrillation: a report of the American college of cardiology/american heart association joint committee on clinical practice guidelines. Circulation 149, e1–e156. 10.1161/CIR.0000000000001193 38033089 PMC11095842

[B15] Kakkar A. K. Mueller I. Bassand J. P. Fitzmaurice D. A. Goldhaber S. Z. Goto S. (2012). International longitudinal registry of patients with atrial fibrillation at risk of stroke: Global anticoagulant registry in the FIELD (GARFIELD). Am. Heart J. 163, 13–19.e1. 10.1016/j.ahj.2011.09.011 22172431

[B16] Kim H. K. Tantry U. S. Smith S. C. Jr. Jeong M. H. Park S. J. Kim M. H. (2021). The East Asian paradox: an updated position statement on the challenges to the current antithrombotic strategy in patients with cardiovascular disease. Thromb. Haemost. 121, 422–432. 10.1055/s-0040-1718729 33171520

[B17] Kuang A. Xu C. Southern D. A. Sandhu N. Quan H. (2024). Validated administrative data based ICD-10 algorithms for chronic conditions: a systematic review. J. Epidemiol. Public Health 72, 202744. 10.1016/j.jeph.2024.202744 38971056

[B18] Larsson K. Janson C. Lisspers K. Jørgensen L. Stratelis G. Telg G. (2013). Combination of budesonide/formoterol more effective than fluticasone/salmeterol in preventing exacerbations in chronic obstructive pulmonary disease: the PATHOS study. J. Intern Med. 273, 584–594. 10.1111/joim.12067 23495860

[B19] Liao K. M. Chen C. Y. (2017). Incidence and risk factors of atrial fibrillation in Asian COPD patients. Int. J. Chron. Obstruct Pulmon Dis. 12, 2523–2530. 10.2147/copd.S143691 28883719 PMC5574688

[B20] Liao K. M. Chen P. J. Chen C. Y. (2023). Prescribing patterns in patients with chronic obstructive pulmonary disease and atrial fibrillation. Open Med. (Wars) 18, 20230864. 10.1515/med-2023-0864 38045860 PMC10693011

[B21] Lin L. Y. Warren-Gash C. Smeeth L. Chen P. C. (2018). Data resource profile: the national health insurance research database (NHIRD). Epidemiol. Health 40, e2018062. 10.4178/epih.e2018062 30727703 PMC6367203

[B22] Linz D. Gawalko M. Betz K. Hendriks J. M. Lip G. Y. H. Vinter N. (2024). Atrial fibrillation: epidemiology, screening and digital health. Lancet Reg. Health Eur. 37, 100786. 10.1016/j.lanepe.2023.100786 38362546 PMC10866942

[B23] Nadeem R. Sharieff A. Tanna S. Sidhu H. Molnar J. Nadeem A. (2015). Potential augmentation of the risk of ischemic cerebrovascular accident by chronic obstructive pulmonary disease in patients with atrial fibrillation. J. Stroke Cerebrovasc. Dis. 24, 1893–1896. 10.1016/j.jstrokecerebrovasdis.2015.04.034 26142261

[B24] Papaporfyriou A. Bartziokas K. Gompelmann D. Idzko M. Fouka E. Zaneli S. (2023). Cardiovascular diseases in COPD: from diagnosis and prevalence to therapy. Life (Basel) 13, 1299. 10.3390/life13061299 37374082 PMC10301030

[B25] Rodríguez-Mañero M. López-Pardo E. Cordero A. Ruano-Ravina A. Novo-Platas J. Pereira-Vázquez M. (2019). A prospective study of the clinical outcomes and prognosis associated with comorbid COPD in the atrial fibrillation population. Int. J. Chron. Obstruct Pulmon Dis. 14, 371–380. 10.2147/copd.S174443 30863038 PMC6388772

[B26] Romiti G. F. Corica B. Mei D. A. Frost F. Bisson A. Boriani G. (2023). Impact of chronic obstructive pulmonary disease in patients with atrial fibrillation: an analysis from the GLORIA-AF registry. Europace 26, euae021. 10.1093/europace/euae021 38266129 PMC10825625

[B27] Simons S. O. Elliott A. Sastry M. Hendriks J. M. Arzt M. Rienstra M. (2021). Chronic obstructive pulmonary disease and atrial fibrillation: an interdisciplinary perspective. Eur. Heart J. 42, 532–540. 10.1093/eurheartj/ehaa822 33206945

[B28] Simons S. O. Heptinstall A. B. Marjenberg Z. Marshall J. Mullerova H. Rogliani P. (2024). Temporal dynamics of cardiovascular risk in patients with chronic obstructive pulmonary disease during stable disease and exacerbations: review of the mechanisms and implications. Int. J. Chron. Obstruct Pulmon Dis. 19, 2259–2271. 10.2147/copd.S466280 39411574 PMC11474009

[B29] Steinberg B. A. Blanco R. G. Ollis D. Kim S. Holmes D. N. Kowey P. R. (2014). Outcomes registry for better informed treatment of atrial fibrillation II: rationale and design of the ORBIT-AF II registry. Am. Heart J. 168, 160–167. 10.1016/j.ahj.2014.04.005 25066554 PMC4145241

[B30] Steinberg B. A. Gao H. Shrader P. Pieper K. Thomas L. Camm A. J. (2017). International trends in clinical characteristics and oral anticoagulation treatment for patients with atrial fibrillation: results from the GARFIELD-AF, ORBIT-AF I, and ORBIT-AF II registries. Am. Heart J. 194, 132–140. 10.1016/j.ahj.2017.08.011 29223431

[B31] Swart K. M. A. Baak B. N. Lemmens L. Penning-van Beest F. J. A. Bengtsson C. Lobier M. (2023). Risk of cardiovascular events after an exacerbation of chronic obstructive pulmonary disease: results from the EXACOS-CV cohort study using the PHARMO data network in the Netherlands. Respir. Res. 24, 293. 10.1186/s12931-023-02601-4 37990197 PMC10662240

[B32] Taiwan Society of Pulmonary and Critical Care Medicine (2018). Taiwan guidelines for the management of pneumonia. Available online at: https://pneumonia.idtaiwanguideline.org/(Accessed June 15, 2025).

[B33] Tsai H. L. Hsiao C. C. Chen Y. H. Chien W. C. Chung C. H. Cheng C. G. (2025). The risk of ischemic stroke in patients with chronic obstructive pulmonary disease and atrial fibrillation. Life (Basel) 15, 0154. 10.3390/life15020154 40003562 PMC11856233

[B34] Van Gelder I. C. Rienstra M. Bunting K. V. Casado-Arroyo R. Caso V. Crijns HJGM (2024). 2024 ESC guidelines for the management of atrial fibrillation developed in collaboration with the european association for cardio-thoracic surgery (EACTS): developed by the task force for the management of atrial fibrillation of the european society of cardiology (ESC), with the special contribution of the european heart rhythm association (EHRA) of the ESC. Endorsed by the european stroke organisation (ESO). Eur. Heart J. 45, 3314–3414. 10.1093/eurheartj/ehae176 39210723

[B35] Wang M. T. Lai J. H. Huang Y. L. Liou J. T. Cheng S. H. Lin C. W. (2021). Comparative effectiveness and safety of different types of inhaled long-acting β_2-_Agonist plus inhaled long-acting muscarinic antagonist vs inhaled long-acting β_2_-Agonist plus inhaled corticosteroid fixed-dose combinations in COPD A propensity score-inverse probability of treatment weighting cohort study. Chest 160, 1255–1270. 10.1016/j.chest.2021.05.025 34023320

[B36] Xiao X. Han H. Wu C. He Q. Ruan Y. Zhai Y. (2019). Prevalence of atrial fibrillation in hospital encounters with end-stage COPD on home oxygen: national trends in the United States. Chest 155, 918–927. 10.1016/j.chest.2018.12.021 30684473

[B37] Yao R. J. R. Andrade J. G. Deyell M. W. Jackson H. McAlister F. A. Hawkins N. M. (2019). Sensitivity, specificity, positive and negative predictive values of identifying atrial fibrillation using administrative data: a systematic review and meta-analysis. Clin. Epidemiol. 11, 753–767. 10.2147/CLEP.S206267 31933524 PMC6712502

