# Supplementary material for: Temporal trends in stroke-prevention medication use in patients with atrial fibrillation and chronic obstructive pulmonary disease
Source: Front Pharmacol. 2026 Feb 18;17:1761934. doi: 10.3389/fphar.2026.1761934 (PMC12957149; doi:10.3389/fphar.2026.1761934)
Supplement: Supplementary file 1 [file Table1.docx]

**Supplementary Table 1. ICD-9-CM and ICD-10-CM codes used to define clinical outcomes**

| **Outcomes** | **ICD-9-CM codes** | **ICD-10-CM codes** |
| --- | --- | --- |
| Ischemic stroke | 362.3, 433.01, 433.11, 433.21, 433.31, 433.81, 433.91, 434.01, 434.11, 434.91, 436 | H34.0, H34.1, H34.2, I63, I67.81, I67.82, I67.89 |
| Intracranial hemorrhage | 430-432 | I60-I62 |
| Gastrointestinal bleeding | 456.0, 456.2, 530.21, 530.7, 530.82, 531.0, 531.2, 531.4, 531.6, 532.0, 532.2, 532.4, 532.6, 533.0, 533.2, 533.4, 533.6, 534.0, 534.2, 534.4, 534.6, 535.01, 535.11, 535.21, 535.31, 535.41, 535.51, 535.61, 537.83, 537.84, 562.02, 562.03, 562.12, 562.13, 568.81, 569.3, 569.85, 569.86, 578 | I85.01, I85.11, K22.11, K22.6, K25.0, K25.2, K25.4, K25.6, K26.0, K26.2, K26.4, K26.6, K27.0, K27.2, K27.4, K27.6, K28.0, K28.2, K28.4, K28.6, K29.01, K29.21, K29.41, K29.51, K29.61, K29.71, K29.81, K29.91, 31.82, K31.811, K55.21, K57.01, K57.11, K57.13, K57.21, K57.31, K57.33, K57.41, K57.51, K57.53, K57.81, K57.91, K57.93, K62.5, K63.81, K66.1, K92.0, K92.1, K92.2 |
